# Comprehensive Study on Ceramic Membranes for Low‐Cost Microbial Fuel Cells

**DOI:** 10.1002/cssc.201501320

**Published:** 2015-12-21

**Authors:** Grzegorz Pasternak, John Greenman, Ioannis Ieropoulos

**Affiliations:** ^1^ Bristol BioEnergy CentreUniversity of the West of EnglandBristolUnited Kingdom; ^2^ Wroclaw University of TechnologyWroclawPoland

**Keywords:** biomass, ceramics, energy conversion, fuel cells, membranes

## Abstract

Microbial fuel cells (MFCs) made with different types of ceramic membranes were investigated to find a low‐cost alternative to commercially available proton exchange membranes. The MFCs operated with fresh human urine as the fuel. Pyrophyllite and earthenware produced the best performance to reach power densities of 6.93 and 6.85 W m^−3^, respectively, whereas mullite and alumina achieved power densities of 4.98 and 2.60 W m^−3^, respectively. The results indicate the dependence of bio‐film growth and activity on the type of ceramic membrane applied. The most favourable conditions were created in earthenware MFCs. The performance of the ceramic membranes was related to their physical and chemical properties determined by environmental scanning electron microscopy and energy dispersive X‐ray spectroscopy. The cost of mullite, earthenware, pyrophyllite and alumina was estimated to be 13.61, 4.14, 387.96 and 177.03 GBP m^−2^, respectively. The results indicate that earthenware and mullite are good substitutes for commercially available proton exchange membranes, which makes the MFC technology accessible in developing countries.

## Introduction

A microbial fuel cell (MFC) is a bio‐electrochemical transducer in which electro‐active bacteria digest organic matter to produce electric current under anaerobic conditions. A typical, widely used MFC design consists of two chambers. Electro‐active bacteria grow in the anodic chamber, in which they oxidise organic compounds and transfer electrons to the anode, and protons are transferred through a proton exchange membrane (PEM) to the cathode compartment. In the cathode, protons combine with incoming electrons from the anode through the circuit and with oxygen from air to form water.^1^ A simplified MFC design consists of an anode chamber and an open‐to‐air cathode.^2^ Despite the simplicity of the air‐cathode systems, an important obstacle in their operation is the need for hydration, particularly if membranes with low molecular weight cut‐off values are used.^3^

The separator between the anode and cathode half‐cells is a crucial component of any MFC, apart from membrane‐less systems.^4^ The membrane and its properties contribute strongly to the mass transport and ohmic losses and limit the voltage and performance of the cell. Moreover, most common designs employ expensive polymeric cation exchange membranes (CEM) because of their high conductivity to protons.^5^, ^6^, ^7^ The resultant need for hydration and the cost of CEMs make them inappropriate for the large‐scale application of air‐cathode‐based systems, in which the simplicity of operation and cost effectiveness play important roles.^8^ The scale‐up of the MFC technology will require the application of low‐cost separators. Materials such as nylon filter, glass fibre mat and non‐woven cloth have been reported previously.^9^

An interesting approach to reduce the cost of MFCs is to build the anode chamber in such a way that the membrane becomes the structural material of the cell.^10^ Such an approach can be used with porous ceramic materials, which so far have been rarely reported as the membranes in MFCs.

The study of Behera et al.^11^ showed that earthenware clay can be employed to build MFCs. The earthenware MFCs, which operated in the fed‐batch mode on synthetic wastewater supplied with sucrose, produced a power density of 1.04 W m^−3^. In their two‐chamber MFCs, operated under continuous‐flow conditions, the current density reached 70.48 W m^−3^, however, a permanganate oxidiser rather than air was used as the catholyte.^12^ Recently, Ghadge and Ghangrekar showed that the performance of ceramic membranes can be increased by 48 % with the addition of a cation exchanger.^13^

Although terracotta is another example of a ceramic material that has been applied successfully,^14^ little effort has been exerted to compare the performance of different ceramic materials in MFCs. Winfield et al.^15^ compared earthenware and terracotta and employed these materials in both cylindrical as well as conventional air‐cathode MFCs. When these cylindrical MFCs (volume of 40 mL) were tested, the earthenware generated a 75 % higher power than the terracotta units. The earthenware membranes appeared to generate similar levels of electricity to CEM.^16^

The comparable power performance of ceramic and polymeric membranes makes the ceramic materials appropriate for application in MFCs. The application of low‐cost ceramic MFCs can be suitable in remote rural areas in developing countries, in which centralised electrical grids or sanitation systems are difficult to provide. The current solutions often involve the combustion of fuels and may lead to the contamination of the environment with hazardous compounds.^17^, ^18^ Thus ceramic MFCs may be a sustainable alternative for electricity generation if a waste product such as urine could be used as a fuel.^19^

For a successful implementation of ceramic membranes in MFCs, the appropriate materials need to be selected. A short‐ or long‐term operation may be required, which depends on the application, as well as the combination of parallel and series connections of multiple units to increase the power output.^3^, ^20^ Nevertheless, no comprehensive comparison of different ceramic membranes is available in the literature. Moreover, pyrophyllite and alumina ceramic tubes have not been tested previously for their functionality in MFCs.

The aim of this study was to compare the performance of four different types of ceramic membranes, mullite, earthenware, pyrophyllite and alumina, and assess their behaviour in cascades of MFCs under continuous‐flow conditions. The MFCs were evaluated for their suitability to use urine as a sustainable fuel. As the membrane is an integral part of the synthetic MFC ecosystem, the impact of ceramic membranes on bio‐film development was also assessed.

## Results and Discussion

### Inoculation stage

**Figure 1:**
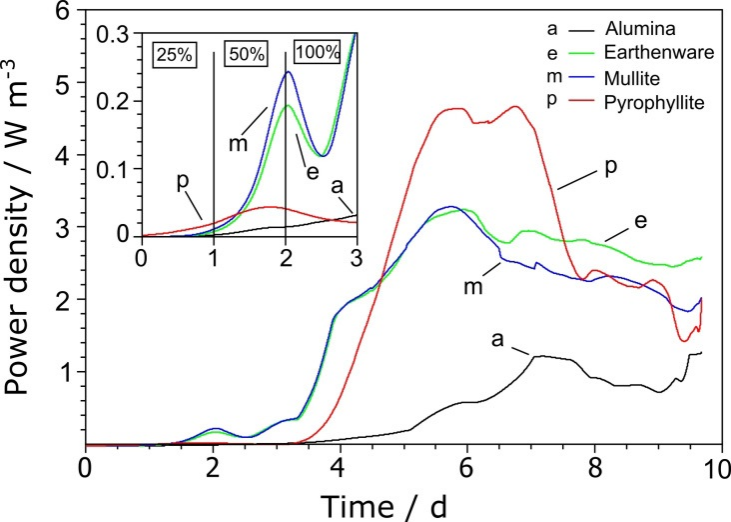
Real‐time power performance monitored over the maturing period of the bio‐film. The bio‐film was matured under continuous‐flow conditions with increasing concentrations of urine (25, 50 and 100 %) with an external load of 2 kΩ. Data represent average values from all cells in the cascades. Inset shows magnified traces over the first 3 days. E=earthenware, M=mullite, P=pyrophyllite, A=alumina.

The enrichment of electro‐active bacteria from anaerobic activated sludge was performed by combining two major selective factors: (i) the maturation of the anodophilic bio‐film under anaerobic conditions with the application of an external load of 2 kΩ and (ii) the increase of the urine/activated sludge ratio in the first days of operation (Figure 1).

As can be seen in Figure 1, the first two days of the inoculation period were characterised by an adaptation phase, in which the urine concentration was 25 %, and a growth phase in which the urine concentration was increased to 50 %; this was clearly marked by the increase in power density. These two phases were clearly observed for earthenware and mullite, whereas for pyrophyllite the increase in power density was less marked. The power output generated from alumina MFCs suggested a slow adaptation of the microorganisms to the electron‐transferring environment. After two days of operation, an initial decrease in power was observed upon switching from wastewater to 100 % urine, which was probably because of the removal of planktonic cells and residual electron mediators. The variety of electron mediators present in the activated sludge has been identified and reported widely.^1^ An increase of the urine concentration to 100 % resulted in the elution of the planktonic bacteria and electron shuttles from MFCs, after which the bio‐film mainly contributed to current production.

The time of operation before the stable phase and high power densities were reached varied between different types of materials used to build the MFCs. For earthenware, pyrophyllite and mullite, relatively stable power output was recorded after 5 days of operation, whereas for alumina this was 7 days. The highest power densities observed during that period for mullite, earthenware, pyrophyllite and alumina were (3.41±0.00), (3.28±0.01), 4.69 and (1.24±0.02) W m^−3^, respectively.

Ceramic MFCs reached the maximum power performance just after 5 days of operation under constant conditions. After this, a significant decrease of power was observed. It is assumed that the decrease of the performance was caused by the rapid decrease of the internal resistance of the MFC whilst the external resistance remained the same.

### Open‐circuit voltage

The maximum and averaged maximum (given in parenthesis) open‐circuit voltage (OCV) values observed during the operation were: 550.0 mV (519.8±13.1 mV) for mullite, 532.3 mV (529.0±2.4 mV) for earthenware, 634.8 mV for pyrophyllite and 482.2 mV (474.6±7.7 mV) for alumina (Table 1). The OCV values observed for earthenware are similar to those observed by other authors.^11^, ^15^

**Table 1:** Performance and cost characteristics of the ceramic membranes, which include values normalised to the volume and anode and cathode surface area. The highest and lowest values are in bold.

Membrane material	*R*_int_ [Ω]	OCV [mV]	Power to volume [W m^−3^]	Power to anode [mW m^−2^]	Power to cathode [mW m^−2^]	Absolute power [μW]	Wall thickness [mm]	Porosity [%]	Cost of membrane [GBP m^−2^]
	min.	max.	max.	max.	max.	max.			
mullite	500	550.0	4.98	3.94	15.44	56.7	4	27	13.61
earthenware	**304**	532.3	6.85	5.43	**32.32**	**78.1**	3.5	14	**4.14**
pyrophyllite	905	**634.8**	**6.93**	**6.16**	25.69	44.4	2	1.8–2	387.96
alumina	2000	499.7	2.60	2.06	11.25	29.7	3	<1	177.03
CMI‐7000S membrane	nd^[a]^	nd^[a]^	nd^[a]^	nd^[a]^	nd^[a]^	nd^[a]^	nd^[a]^	nd^[a]^	79.17

[a] Not determined.

### Polarisation experiments

**Figure 2:**
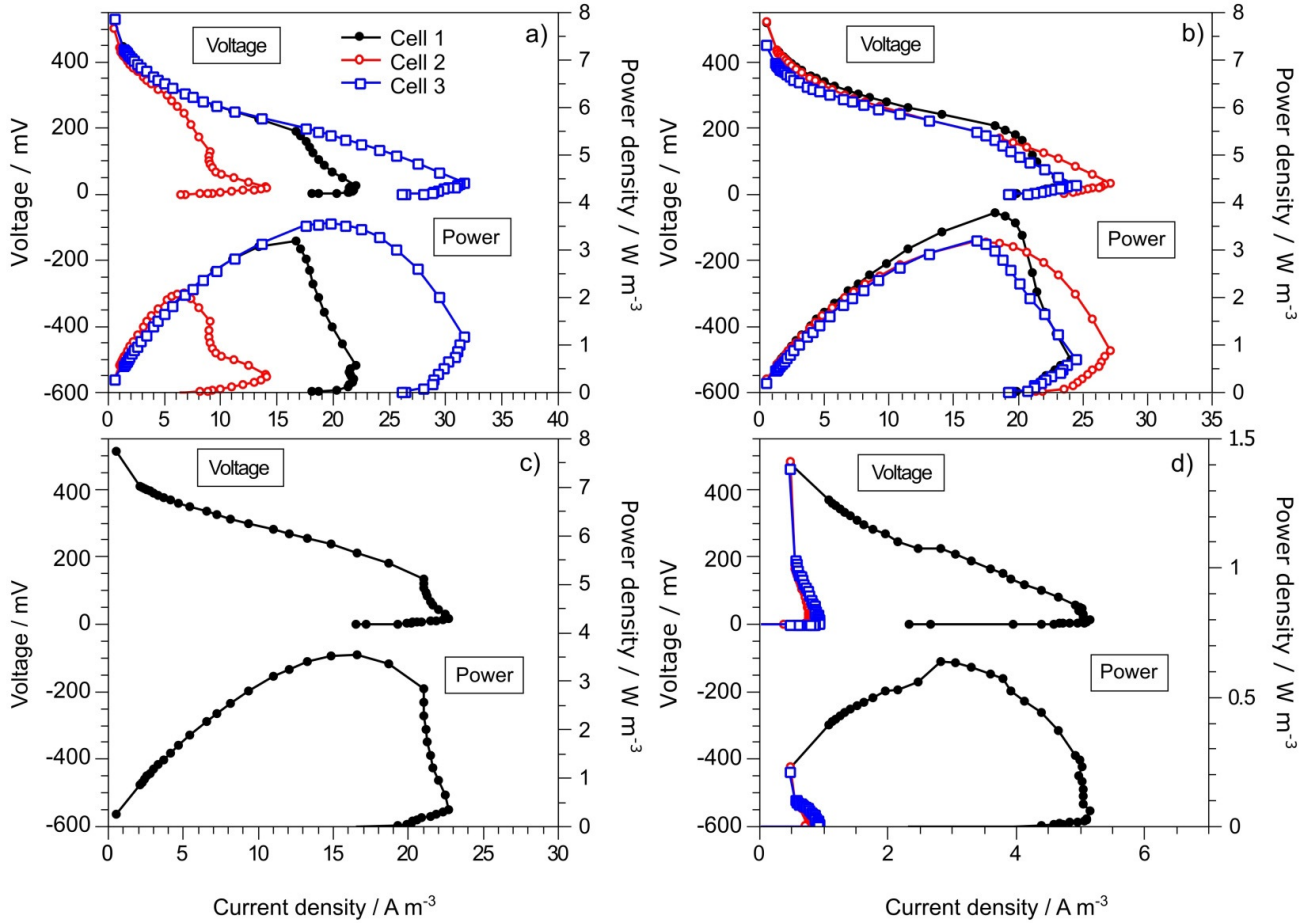
Polarisation (top) and power density curves (bottom) to compare the performance of different ceramic MFCs with immature bio‐film 10 days after inoculation. Data for: a) mullite, b) earthenware, c) pyrophyllite, d) alumina. Cell 1, Cell 2 and Cell 3 represent the first, second and third MFCs in the cascade, respectively. Power and current densities were normalised to the total volume of the MFCs.

Polarisation experiments were performed throughout the experimental period with an interval of approximately one week during the first month of operation. Data obtained from polarisation experiments after 10 days of operation, normalised to the total bio‐reactor volume, are presented in Figure 2.

### Double overshoot phenomenon

In all of the cells, an overshoot phenomenon was observed. The overshoot phenomenon occurs at high current densities and is thought to be a function of internal resistance. The demand for electrons overcomes the metabolic abilities of microorganisms and decreases the electron‐transfer rate as a result of the depletion of electron‐transfer compounds (ETCs) in the anodic chamber.^21^ Its occurrence has been commonly reported as a consequence of anodic reactions^22^ and can be controlled by adapting the bio‐film to higher anode potentials.^23^

In this study, the overshoot observed after 10 days of operation was clearly a result of an immature bio‐film. A double overshoot was observed in the first and second mullite cells. When the current density reached 9.05 A m^−3^ (cell 2), the subsequent decrease of the external load value resulted in a concomitant decrease in the current density, following which an increase of the current density was observed. The later decrease of external load caused the occurrence of another overshoot. An explanation for this current increase was proposed by Ieropoulos et al.^21^ who suggested that the dynamic adjustment of the microbial community, which adapt to the difficult conditions, was responsible for the increase of current. The occurrence of a double overshoot indicates that the anodophilic bacteria were able to overcome a moderate demand for current but could not overcome the highest demand at this stage of bio‐film development.

### Adverse effect of membrane type on bio‐film development

Although mullite and alumina MFCs produced diverse ranges of current, relatively uniform characteristics of power curves were observed for earthenware MFCs if we compare the individual MFCs within the cascade (Figure 2). The mullite MFCs generated 22.0 (cell 1), 9.05 (cell 2) and 31.5 A m^−3^ (cell 3). A similar pattern was observed for the power output. The polarisation curves indicated substantial mass transfer losses in the first two cells in the cascade. Additionally, pyrophyllite and the first earthenware MFCs revealed significant mass transfer losses. Substantial differences in current and power densities were observed among alumina MFCs, in which the first cell generated the highest power output (0.67 W m^−3^) and only negligible current generation was observed for the two other cells in the cascade. This variance was not observed for the earthenware MFCs. The maximum current densities in the earthenware MFCs ranged between 24.04 and 27.09 A m^−3^, and the maximum power densities ranged between 3.18 and 3.89 W m^−3^. The pyrophyllite MFC generated a maximum current density of 22.69 A m^−3^ and a maximum power density of 3.21 W m^−3^.

The power characteristics of the individual MFCs of one type become more uniform with time because of further bio‐film development (Figure S2). These results suggest that the dynamic behaviour of bio‐film development was highly dependent on the type of membranes used to build the MFCs. The term “dynamic behaviour” refers to the kinetics of the bio‐film growth, which is subject to numerous factors and governed by Monod's microbial growth principles. Apart from pyrophyllite, all of the cells were designed to maintain similar hydrodynamic conditions. Nevertheless, all of the membranes varied in composition, density and porosity.

The hypothesis for the observed patterns after 10 days of MFC operation is related to the simultaneous adverse effect of mass transfer and the physicochemical changes of the feedstock on the development of microbial communities. The higher rate of protons that pass through a particular membrane type encourages electro‐active microorganisms to develop on the anode surface. In contrast, the membranes characterised by a lower performance created an antagonistic environment in the anode chamber. This effect was strengthened by oxygen diffusion through the more porous mullite membranes, which promoted the growth of facultative aerobic species and possible substrate diffusion through the membranes (Chae et al.^6^), and by consequent changes in the feedstock physicochemical composition at different stages of treatment in the cascades.

The polarisation experiments suggest that the bio‐film and its performance were highly affected by the type of membrane used to build the MFCs. Only a negligible overshoot phenomenon was observed after 18 days of operation for earthenware and pyrophyllite. For mullite and alumina, more symmetric polarisation curves were recorded after 32 days of operation (Figure S2). The results revealed that the most favourable conditions for the early stage of the development of electro‐active microorganisms were created in MFCs by using earthenware and pyrophyllite membranes. This underlines the importance of the appropriate material selection to obtain homeostasis in synthetic ecosystems such as MFCs. Thus the development of appropriate microbial consortia will be highly dependent on the type of material used as a membrane.

### Comparison of ceramic membranes and their physical properties

**Figure 3:**
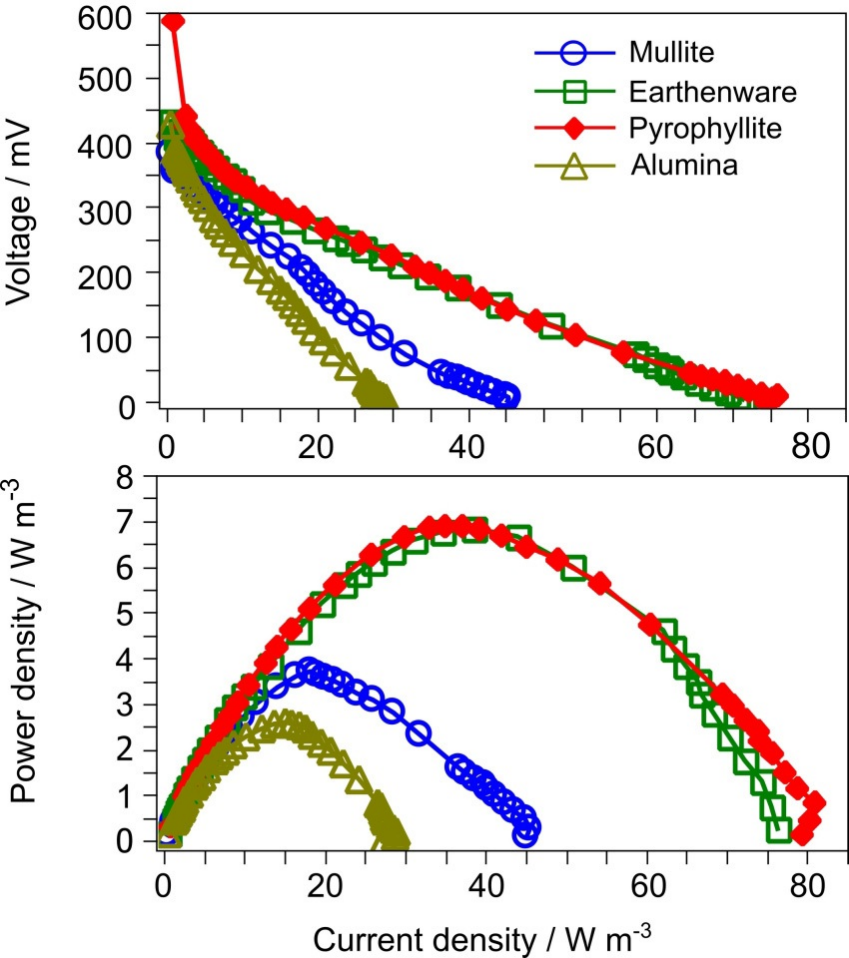
Polarisation and power density curves for the first MFCs in the cascades after 32 days of operation. Values were normalised to the total volume of MFCs.

The analysis of data obtained from the polarisation experiment after 32 days of operation (Figure 3) confirmed that both earthenware and pyrophyllite membranes out‐performed the rest. Only one month of operation was sufficient to reduce the mass transfer loses and overshoot phenomenon in all types of materials. The polarisation curves for earthenware and pyrophyllite MFCs are almost identical; however, significant activation loses occurred in the case of pyrophyllite. These activation loses are observed at low current densities. The OCV observed for pyrophyllite was 589.3 mV, whereas the application of a 30 kΩ load resulted in a 25 % voltage drop to 443.2 mV.

The activation losses are caused by several factors that include bio‐film resistivity, charge transfer to anode, catalysts, reactants, type of electrode, bacterial species or operational conditions.^1^, ^5^, ^24^ The observation of such a high decrease of voltage was probably a result of the type of substrate supplied to MFCs or affected by the lower hydraulic retention time (HRT) in pyrophyllite cells (Table S1). The higher HRT may lead to the elution (from the MFCs) of biologically synthesised ETCs, biomass and other biomolecules that contribute to power generation. According to Li et al.,^25^ higher HRTs contributed to higher chemical oxygen demand (COD) removal and a higher power generation. Another study showed that HRT had an impact on the internal resistance of the cell, and this effect was dependent on the type of inoculum and the stage of bio‐film development.^21^

With regard to the mass transfer losses, pyrophyllite was a better‐performing material. In terms of power density, pyrophyllite and earthenware gave similar results and generated power densities of 6.93 and 6.86 W m^−3^, respectively.

The observed differences in the generated current for all of the materials were mainly affected by their porosity and thickness (Table 1). The thickness affected the distance between the two electrodes, which has an influence on the internal resistance of the cell.^26^, ^27^ The relatively low performance of the mullite cells could be affected by the thickness, high porosity (27 %) and consequent oxygen back diffusion as well as the excessive transport of anolyte components to the cathode. Furthermore, the diffusion of anyolite could decrease the oxygen diffusion to the cathode surface.

The worst‐performing material was alumina (power output of 2.63 W m^−3^, current density of 28.3 A m^−3^). In contrast to highly porous mullite, the low porosity of the alumina membranes (below 1 %) may have inhibited sufficient proton transfer.

A compromise between the wall thickness and porosity is a matter of great importance. In this study, such a compromise was found in the pyrophyllite‐based MFCs (1.8–2.0 %, 2 mm), regardless of the crystal structure and composition, which certainly also affects the transport of protons and other ionic species.

### Short‐term performance

**Figure 4:**
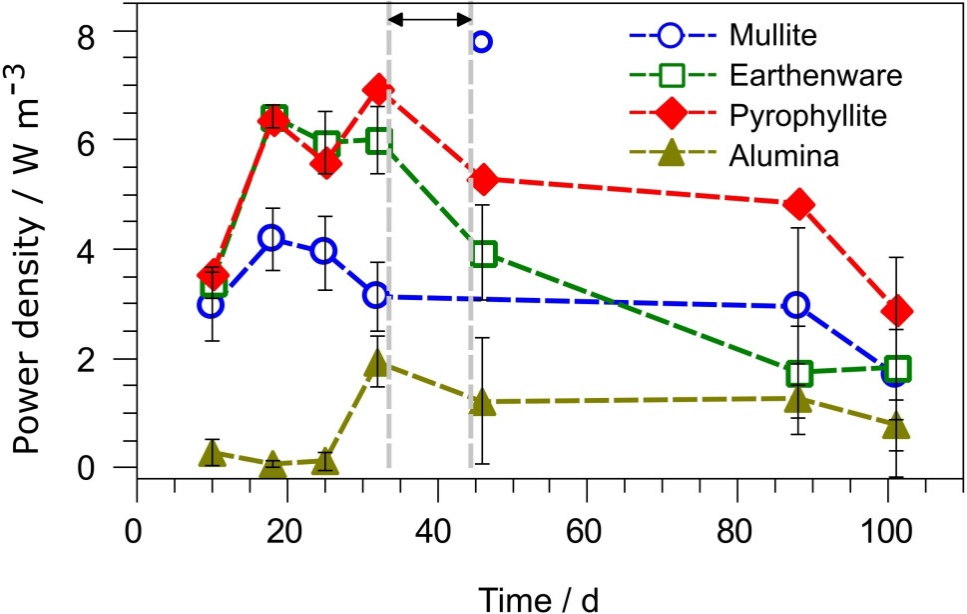
Maximum power points extracted from polarisation experiments over the whole experimental period. Data points represent the average values from three replicates and error bars represent standard deviations. The arrow indicates the period in which the energy‐harvesting experiment was performed. The error bars for the outstanding value (mullite, 46 days, explanation in text) were removed for clarity.

To determine the effect of the short‐ and long‐term operation of MFCs on the membrane performance, the maximum power points were extracted from polarisation experiments, and the results are shown in Figure 4. The short‐term operation was studied for 32 days, after which the cells were subjected to an energy‐harvesting experiment. The energy‐harvesting experiment was performed between days 33 and 44 of operation and its purpose was to simulate the practical implementation scenario (data not shown).

The short‐term operation revealed interesting differences between the materials. The pyrophyllite and earthenware performances were almost identical. Earthenware MFCs obtained a maximal power density of (6.43±0.21) W m^−3^ after 18 days of operation, whereas the highest performance for pyrophyllite was observed after 32 days of operation (6.93 W m^−3^). In the case of mullite, 18 days was also sufficient to reach the maximum power density of (3.30±0.45) W m^−3^. A different pattern was observed for alumina membranes, for which the highest power output was observed after 32 days of operation and reached (1.50±0.49) W m^−3^.

The highest performance was observed in MFCs supplied with membranes with porosities from 2 to 14 % (Table 1). A possible explanation for why the alumina membranes produced a significant amount of power after a long lag‐period is that its low porosity inhibited the water diffusion from the anode chamber and consequent hydration of the cathode. Once sufficient water molecules were produced on the cathode, a subsequent increase of proton transfer occurred as a result of an electro‐osmotic drag mechanism, that is, “dragging” the water molecules and potentially the ions from the anolyte to the cathode surface.^28^, ^29^

### Long‐term performance

To verify if the ceramic MFCs could be used successfully for energy‐harvesting purposes, the cells were connected as a stack in a series–parallel configuration and the MFCs were used to power a magnetic stirrer. During this experiment, cell reversal was observed. After this experiment, a significant decrease of the power density was recorded for all types of material. The decrease of the power density observed at that stage for all of the materials was probably caused by the sub‐optimal load applied and subsequent cell reversal observed in the MFCs that led to a decrease of the MFC performance. The power density of the first cell in the cascade increased by 352 % and reached 10.4 W m^−3^. However, this effect was only temporary. Therefore, it is assumed that the increased power output in this cell may have resulted from the capacitance of mullite.^30^, ^31^, ^32^

The power density for earthenware after 88 days was 1.4 W m^−3^, which corresponds to a power drop of 72 % from the initial value. The power density generated in pyrophyllite MFCs decreased by 23 % to 4.3 W m^−3^, by 30 % to 2.3 W m^−3^ (mullite) and by 33 % to 1.0 W m^−3^ for alumina. During the last days of operation, significant amounts of salts were observed on the cathode, which confirms that electro‐osmotic drag and diffusion had taken place. These salts could inhibit proton transfer by limiting the rate of oxygen reduction.^33^ Behera et al.^11^ noticed a power performance decrease in earthenware MFCs, explained by the increase of bio‐film growth on the cathode and a similar decrease is observed commonly for enzymatic bio‐fuel cells.^34^ Winfield et al.^16^ reported an increase in power over time for earthenware MFCs that were run as fed‐batch cultures, which reached a power density of 4.5 W m^−3^ after 7 months of operation, whereas in this study a power density of above 6 W m^−3^ was noticed only 18 days after inoculation.

The electron‐generating activity of a bio‐film is likely to peak over time, particularly if the mixed bacterial consortia colonize the anode surface.^35^ The thickness of the bio‐film also affects its conductivity and the substrate diffusion.^24^ Another dynamic element in MFCs is the fouling and bio‐fouling of the membranes.^7^ It is suggested that the power decrease in ceramic MFCs may be the result of combined biological, physicochemical and electrochemical reactions that take place, which include the aging of the bio‐film, changes of its thickness, the precipitation of uric salts, accumulation of salts on the cathode and its possible deterioration, and the growth of microorganisms on the highly porous membranes.

### Microbiological and physicochemical analysis

The COD of fresh urine was measured over the experimental period as (5862±760) mg${}_{O{}_{2}}$ l ^−1^. The COD removal for the tested materials reached (41.5±5.9) % for mullite, (46.5±4.5) % for earthenware, (50.2±3.7) % for pyrophyllite and (49.4±7.3) % for alumina. The results of a t‐test (*α*=0.05) showed no significant difference (*p*>0.05) between the COD removal by all types of MFCs. Surprisingly, the worst‐performing MFCs built from alumina showed as high a COD removal as the other materials. It is assumed that other competitive reactions took place in the MFCs, which include fermentation or the aerobic growth of heterotrophs, and that the urea, a major constituent of urine, did not contribute to power generation because of its hydrolysis by ureases. The pH and conductivity of neat urine increased after treatment in MFCs. The pH increased from pH 6.10–6.97 to 8.80–9.05, whereas the conductivity increased from 11.80–13.40 to 18.48–19.32 mS. This increase was mainly caused by the hydrolysis of urea.

In several other studies, the bacterial cell production rate was correlated positively to the power generated by MFCs, however, pure bacterial cultures were investigated in the majority of these studies.^36^ If the bacterial consortia obtained by enrichment from activated sludge were used as a source of electrogens, a significant amount of biomass was reported in open‐circuit bio‐reactors.^37^ In such diverse microbial populations, a substantial part will not contribute to current generation.

**Figure 5:**
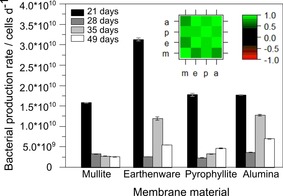
Bacterial biomass collected for each type of MFC. The insert represents correlogram, m=mullite, e=earthenware, p=pyrophyllite, a=alumina.

A correlation was found when the bacterial counts for the different materials were compared. The correlation coefficients between all types of MFCs were >0.85 (Figure 5, inset). In all cases, the biomass reached the highest values in the early stage of bio‐film development. The number of daughter cells varies across each stage of bio‐film development.^38^ A possible reason for this outstanding activity is ongoing bio‐film formation, in which bacterial species colonise the anode and compete for their ecological niche. After the maximum was reached, the cell divisions stabilised over time, and a decrease of the bacterial production rate was observed. Although all of the MFCs produced the same order of magnitude of biomass, the highest number of cells was observed for earthenware. The monitoring of the amount of biomass suggested that the bacterial cell numbers reflect the state of bio‐film development, and the highest number of bacterial cells was noticed in the early stage of colonisation.

### Composition and structure of ceramic membranes

**Figure 6:**
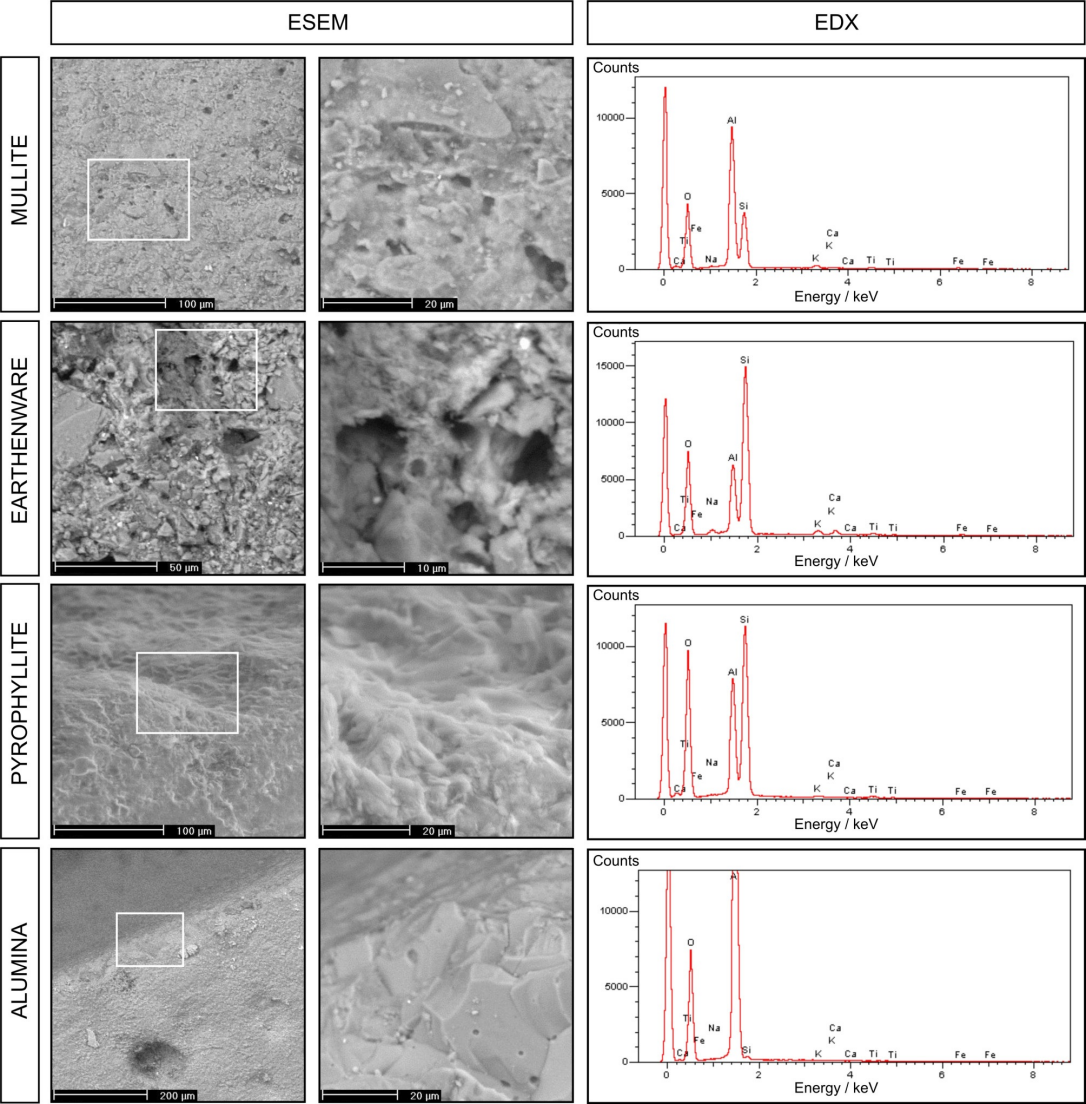
ESEM micrographs and EDX spectra that show the topography and elemental composition of the ceramic membranes. The middle column of the micrographs represents a higher magnification of the region marked by the white rectangle (first column).

The microstructure of ceramic membranes varied as well as its elemental composition (Figure 6). Mullite and earthenware revealed irregular and porous structures. The density of the pore distribution was higher in mullite. Moreover, the topography of this material was more uniform. Large >10 μm macropores were observed both in earthenware and mullite membranes. The integrity of pyrophyllite and alumina membranes was very high in comparison to earthenware and mullite, and only small <1 μm pores were observed in the alumina membranes.

The primary elements of all of the ceramic membranes were Si and Al. The chemical composition was estimated from the stoichiometric ratio of each individual element (Table 2). Interestingly, two of the materials, earthenware and pyrophyllite, which had the highest power performance in this study, showed a similar concentration of SiO_2_: 67.92 % for earthenware and 64.54 % for pyrophyllite. A similar concentration of Al_2_O_3_ was also noticed for these materials: 22.00 % for earthenware and 32.96 % for pyrophyllite. Moreover, their abundance in the chemical composition was also the highest and substantial amounts of other compounds were recorded. Whether the elements present in these ceramic membranes play a role in cation transfer in MFCs requires further study.

**Table 2:** Chemical composition of ceramic membranes estimated by the stoichiometric ratio of oxygen and other elements.

Compound	Composition [%]			
	mullite	earthenware	alumina	pyrophyllite
Al_2_O_3_	59.72	22.00	97.69	32.96
CaO	–^[a]^	2.88	–^[a]^	0.21
Fe_2_O_3_	1.01	0.75	–^[a]^	–^[a]^
K_2_O	0.93	1.24	–^[a]^	0.33
MgO	–^[a]^	0.57	–^[a]^	–^[a]^
Na_2_O	–^[a]^	2.73	–^[a]^	–^[a]^
P_2_O_5_	–^[a]^	‐^[a]^	0.27	0.61
SiO_2_	36.65	67.92	2.02	64.64
SO_3_	–^[a]^	0.84	–^[a]^	–^[a]^
TiO_2_	0.97	0.94	–^[a]^	0.96

[a] Not detected or below the detection limit.

### Cost analysis

The cost comparison (Table 1) was based on the UK market. However, the earthenware was imported from India. Despite the freight costs, this material was the cheapest one for tubular ceramic MFCs. Its cost was estimated to be only 4.14 GBP m^−2^. This is in contrast to pyrophyllite, the material that revealed a comparable power performance, but better long‐term performance, the price of which was estimated to be 387.96 GBP m^−2^. The other material that was applied successfully as a membrane in MFCs was mullite (13.61 GBP m^−2^), which had a moderate short‐term performance in comparison to earthenware and pyrophyllite.

The price of earthenware and mullite makes them good substitutes for polymeric membranes, the cost of which is estimated to be 79.17 GBP m^−2^ (CEM). The price of pyrophyllite is disproportionate to any performance advantages (Table 1) that are likely to be gained from its application, which is also the case for alumina. However, both of these ceramics remain valid materials to investigate the role of structure, composition and properties in proton transfer in MFCs.

## Conclusions

Different types of ceramic materials have been employed successfully as proton exchange membranes in single‐chamber microbial fuel cells (MFCs). The time needed to reach the maximum power for ceramic MFCs was estimated to be between 18 and 32 days of operation, which proves the favourable conditions for bio‐film development in comparison to polymeric membranes. The microbial activity reflected the feedback between the bio‐film and the type of membranes used, which underlines the importance of appropriate MFC design to achieve homeostasis in synthetic ecosystems. The maximum power was generated by earthenware and pyrophyllite systems, however, long‐term operation revealed similar levels of performance between earthenware and mullite.

The long‐term operation was affected by an increase of the internal resistance, probably as the result of the increasing thickness of the bio‐film, the bio‐fouling of porous materials and the precipitation of uric salts, which may have limited the diffusion of the nutrients. The analysis of cost and performance indicated that ceramic materials could be employed successfully in large‐scale applications. However, further studies are needed to determine the role of long‐term operation on the properties and performance of these materials. It has been shown that the performance of the MFCs was related to the physical and chemical properties of the ceramic membranes. The membranes with the highest porosity and the highest concentration of silica showed the highest power performance.

## Experimental Section

### MFC construction

Single‐chamber continuous‐flow MFCs (SCMFCs) were built by using ceramic cylinders as proton exchange membranes, which were cut to length to maintain the same internal volume. Four types of materials were used: mullite EM80P (Anderman Industrial Ceramics, UK), earthenware (Scientific&Chemical Supplies Ltd, UK), unfired pyrophyllite and alumina (Ceramic Substrates and Components Ltd, UK). The internal volume of the empty MFCs was 11.4 mL, apart from the MFC composed of pyrophyllite, which was supplied in a pre‐defined 6.4 mL volume. The cells were supplied with carbon veil anodes and conductive paint cathodes along with a nickel‐chromium mesh as a current collector. The anodes were prepared from unmodified carbon veil with a density of 20 g m^−2^ (PRF Composite Materials, Dorset, UK). The carbon veil was folded around a plain NiCr wire (Ø 0.45 mm, Scientific Wire Company, UK), cut and wrapped around the axis to form a brush‐type shape with a total surface area of 144 cm^2^. Air cathodes were prepared by covering the outer ceramic surface with conductive graphite paint. Graphite paint was prepared as described by Winfield et al.^39^ Briefly, polyurethane rubber coating (PlastiDip, Petersfield, UK) was dissolved in petroleum spirit and mixed with graphite (Fisher Chemicals, UK) in a 2:3 (plastidip/graphite) ratio. A NiCr wire mesh (20×20, 0.18 mm) was used as the current collector. The cathode surface was different for all of the types of materials used in this study because of differences in the wall thickness and ceramic porosity. The projected surface area was 36.74 cm^2^ for mullite, 17.27 cm^2^ for pyrophyllite, 24.18 cm^2^ for earthenware and 26.38 cm^2^ for alumina. The full physical characteristics of the MFCs are given in Table S1.

Each cell comprised a transparent acrylic lid (3 mm thick), as well as a 3 D‐printed acrylonitrile butadiene styrene (ABS) lid with inlet and outlet tubes (Figure S1). The ABS lids were designed in SolidWorks 2013 software and manufactured by using a Fortus 250 mc 3‐d printer (Stratasys, Israel). The ABS lid that covered the top part of the MFC was used to separate the anodic chamber into two smaller sections. The MFC inlet introduced the fuel stream into one compartment, and the outlet was placed in the second section. These hydrodynamic conditions were maintained to help to achieve sufficient flow diffusion rates and biomass removal. Both lids were attached to the ceramic membranes by a single plain nylon screw (Ø 3 mm, RS, UK). Silicon gaskets were used to seal the space between the acrylic and ABS lids.

### Experimental set‐up

Each MFC ceramic material was used in triplicate, apart from pyrophyllite, which was the most expensive option. As a material, it would not meet the low‐cost criteria but it was still interesting to test even as a single unit. The cells were connected as cascades and isolated fluidically by a gas‐gap fluid‐drip mechanism to avoid a liquid electrical conductive bridge between them.

### MFC inoculation and operation

The MFCs were inoculated in a continuous‐flow regime. To enrich the electro‐active bacterial population able to utilise urine, anaerobic activated sludge (750 mL, Wessex Water, Saltford, UK) was mixed with fresh human urine. The urine concentration was increased every day, and the starting concentration was 25, 50 and 100 % (v/v) for the first, second and third day, respectively. During this inoculation period, all MFCs worked under a 2 kΩ external load. After 10 days of operation, polarisation experiments were performed to match the internal resistance of the MFCs. Therefore, 400, 700, 1200 and 2000 Ω was applied to the earthenware‐, mullite‐, pyrophyllite‐ and alumina‐based MFCs, respectively.

Each cascade was fed continuously by using a multi‐channel peristaltic pump (Watson Marlow, USA) at a constant flow rate of 0.35 L d^−1^. Fresh human urine was used as a fuel. Urine was collected from healthy individuals and pooled daily. Before treatment, several pooled urine samples were mixed and acclimated to RT. Mixed urine was routinely analysed in terms of COD, pH and conductivity. The COD was analysed in urine samples before and after treatment in MFCs. The COD test kit was used according to the manufacturer's instructions (Camlab, UK) by using an MD200 colorimeter (Camlab, UK).

### Flow cytometry

The bacterial cell counting was performed by flow cytometry. Samples that contained bacterial cells were collected from the outlet of the MFCs, micro‐centrifuged (12 000 rpm, 1 min) and washed twice with NaCl solution (0.85 %). All samples were diluted in 0.2 μm filtered NaCl solution to reach a concentration that did not exceed 10^6^ cells mL^−1^. Diluted samples were stained with BacLight Green Bacterial Stain according to the manufacturer's instructions (Life technologies, USA).

Flow cytometry measurements were performed by using a BD Accuri C6 flow cytometer (BD, USA). Diluted samples were delivered to an interrogation point at a low flow rate (14 μL min^−1^). Side scatter (SSC), forward scatter (FSC) and green fluorescence signals were measured. Green fluorescence (FL1) was collected by using a 533 band‐pass filter (518–548 nm). The threshold was set to the FSC signal. The discrimination of bacteria and noise generated by the apparatus was performed by a combination of FSC and SSC signals and filtered NaCl solution as the control sample. Gating for the combined FL1 and FSC signals was set up, and the data were processed and analysed.

### Polarisation experiments

Polarisation experiments were performed by using a fully automated variable resistor system.^40^ The range of resistors for the polarisation run was 1 MΩ to 3.75 Ω. The sampling interval was 5 min for each resistance value. The same procedure was repeated several times over the experimental period (Figure 4).

### Environmental electron scanning microscopy and energy dispersive X‐ray spectroscopy

The microstructure and elemental composition of ceramic membranes was investigated by field‐emission environmental electron scanning microscopy and energy dispersive X‐ray spectroscopy (ESEM/EDX; Philips XL‐30). Before analysis, the ceramic membranes were washed with distilled water and dried at 100 °C.

### Data logging

The performance of the MFC was recorded by using an Agilent 34972 A Data Acquisition unit (Agilent Technologies, USA) with a sampling rate of 3 min. The current was calculated according to Ohm's law: *I*=*V*/*R*, in which *V* is the measured voltage [V] and *R* is the value of the external resistance [Ω]. The power output *P* [W] was subsequently calculated using the equation: *P*=*I*×*V*.

### Data processing and statistical analysis

Experimental data were processed by using Microsoft Excel 2010 and plotted by using SciDAVis (v. D001) software. Data collected from flow cytometry experiments were processed with Flowing Software (v. 2.5.1). The statistical analysis was performed with the R Gui (v. 3.1.2) statistical environment. Statistical analysis consisted of the determination of Pearson's correlation coefficients, standard deviations, Shapiro–Wilke's normality test and a t‐test (*α*=0.05).

## Supporting information

As a service to our authors and readers, this journal provides supporting information supplied by the authors. Such materials are peer reviewed and may be re‐organized for online delivery, but are not copy‐edited or typeset. Technical support issues arising from supporting information (other than missing files) should be addressed to the authors.

Supplementary
