# Supplementary material for: Comprehensive Study on Ceramic Membranes for Low‐Cost Microbial Fuel Cells
Source: ChemSusChem. 2015 Dec 21;9(1):88–96. doi: 10.1002/cssc.201501320 (PMC4744959; doi:10.1002/cssc.201501320)
Supplement: Supplementary file 1 — Supplementary [file CSSC-9-88-s001.pdf]

CHEMISTRY & SUSTAINABILITY
CHEMSUSCHEM
ENERGY & MATERIALS

Supporting Information

Comprehensive Study on Ceramic Membranes for Low-Cost Microbial Fuel Cells

Grzegorz Pasternak,[a, b] John Greenman,[a] and Ioannis Ieropoulos*[a]

cssc_201501320_sm_miscellaneous_information.pdf

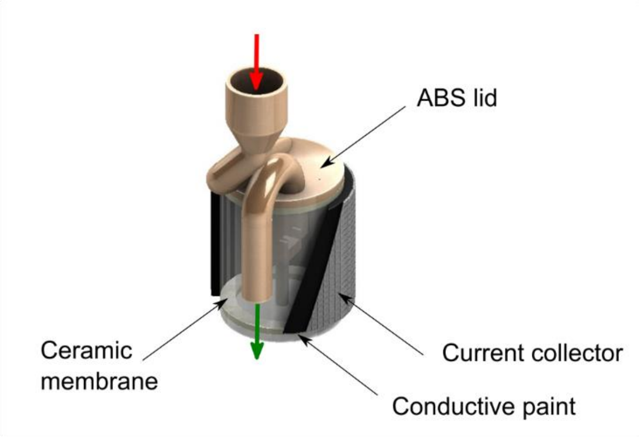

Figure S1. Schematic representation of MFC design. Bold arrows represent inlet and outlet.

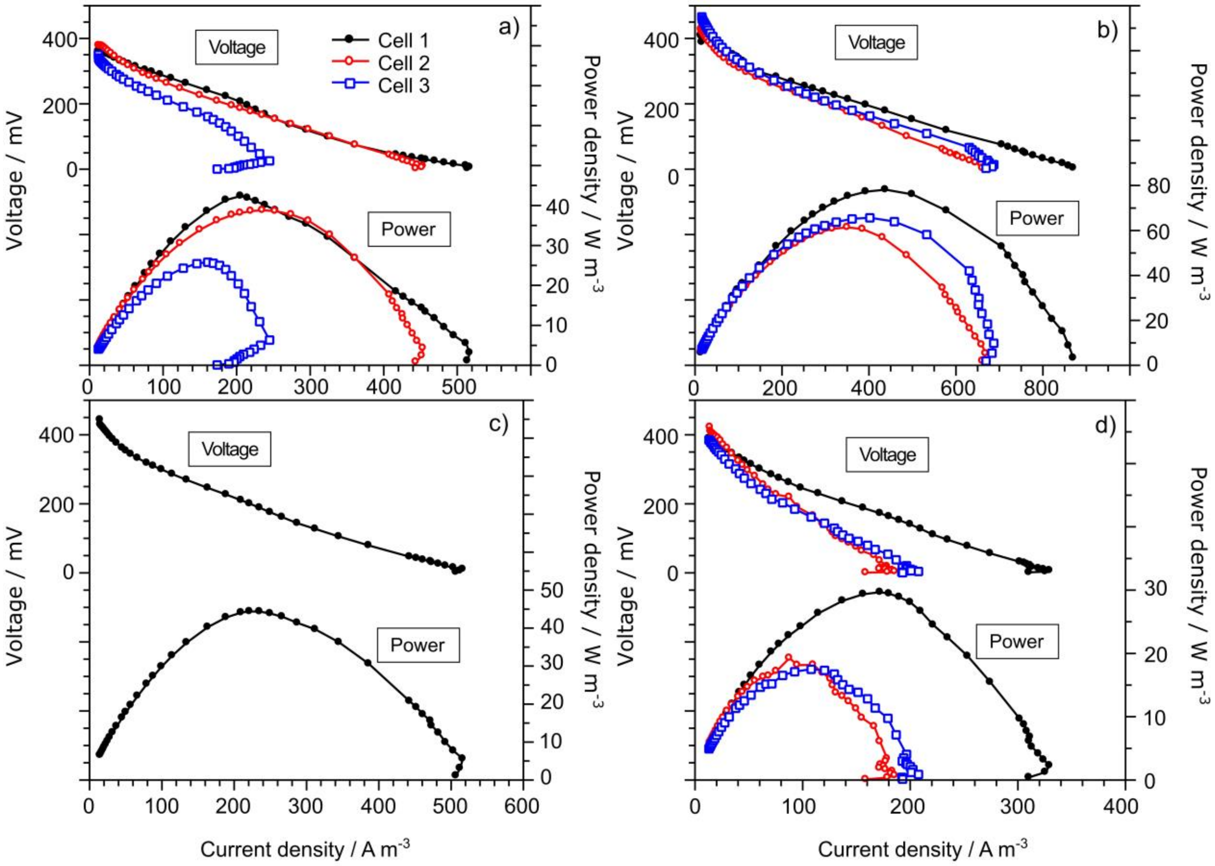

Figure S2. Polarization (top) and power density curves (bottom) comparing the performance of different ceramic MFCs with immature biofilm, after 32 days from inoculation. Data for: a) mullite, b) earthenware, c) pyrophyllite, d) alumina. Cell 1, Cell 2 and Cell 3 represents the first, second and third MFC in the cascade, respectively. Power and current densities were normalized to the total volume of MFCs.

Table S1. Characteristics and cost comparison of ceramic microbial fuel cells and ceramic membranes.

Material	Calculated volume (empty) [mL]	Hydraulic retention time [h]	Cathode surface area [cm^{-2}]	Anode surface area [cm^{-2}]	Cost of unit [GBP]
Mullite	11.4	0.63	36.74	144	0.50
Earthenware	11.4	0.63	24.18	144	0.10
Pyrophyllite	6.4	0.35	17.27	72	6.70
Alumina	11.4	0.63	26.38	144	4.67
